# Time Utilization Among Immunization Clinics Using an Electronic Immunization Registry (Part 2): Time and Motion Study of Modified User Workflows

**DOI:** 10.2196/39777

**Published:** 2023-03-16

**Authors:** Samantha B Dolan, Rachel Wittenauer, Anne Njoroge, Penina Onyango, George Owiso, Jessica C Shearer, William B Lober, Shan Liu, Nancy Puttkammer, Peter Rabinowitz

**Affiliations:** 1 International Training and Education Center for Health University of Washington Seattle, WA United States; 2 Department of Global Health University of Washington Seattle, WA United States; 3 Bill and Melinda Gates Foundation Seattle, WA United States; 4 County Department of Health Siaya Kenya; 5 International Training and Education Center for Health University of Washington Nairobi Kenya; 6 PATH Seattle, WA United States; 7 Biobehavioral Nursing and Health Informatics University of Washington Seattle, WA United States; 8 Department of Industrial and Systems Engineering University of Washington Seattle, WA United States

**Keywords:** immunizations, electronic immunization registry, workflow, time and motion, digital health intervention, mixed methods evaluation

## Abstract

**Background:**

Digital health interventions have the potential to improve the provision of health care services through digitized data collection and management. Low- and middle-income countries are beginning to introduce electronic immunization registries (EIRs) into their routine immunization services to better capture and store childhood vaccination information. Especially in Africa, where 25% of children remain unimmunized or underimmunized, technologies that can help identify children due for a vaccination are particularly important for improving vaccination coverage. However, an improved understanding of the effectiveness of these systems is needed to develop and deploy sustainable EIRs in low- and middle-income countries.

**Objective:**

We conducted an interventional pretest-posttest design study that sought to improve time efficiency through workflow modifications in Kenyan immunization clinics. Our aim was to describe how activity times differed after introducing workflow modifications that could potentially reduce the time needed to perform routine data entry activities. Our intent was to demonstrate changes in efficiency when moving from the existing dual–data entry workflow to a future paperless workflow by health facility size and experience length of health care workers (HCWs).

**Methods:**

We tested how 3 workflow modifications would affect time utilization among HCWs using the EIR at the point of care compared with baseline immunization clinic workflows. Our outcome of interest was the time taken to complete individual activities and a patient’s total time in the clinic where we compared the time spent during the baseline workflow with that during the modified workflow. We used a standardized tool to observe and document the immunization clinic workflow. To estimate differences in time utilization, we used bivariate analyses and fit multivariate linear mixed-effects models.

**Results:**

Our study found that for HCWs using an EIR, the introduction of modified workflows decreased the amount of time needed to provide services to children seen in the immunization clinic. With a baseline mean time of 10 minutes spent per child, this decreased by about 3 minutes when the preparation modification was introduced and almost 5 minutes for the paperless and combined modifications. Results pertaining to the EIR’s performance and ability to connect to the internet were particularly insightful about potential causes of delays.

**Conclusions:**

We were able to conduct a concise clinical simulation exercise by introducing modified workflows and estimating their impact on time utilization in immunization clinics using an EIR. We found that the paperless workflow provided the largest time savings when delivering services, although this was threatened by poor EIR performance and internet connectivity. This study demonstrated that not only should digital health interventions be built and adapted for particular use cases but existing user workflows also need to adapt to new technology.

## Introduction

### Background

Digital health interventions (DHIs) have the potential to improve the provision of health care services. Through digitized data collection and management, these interventions can improve the accessibility and use of patient information, support clinical decisions, and improve communication between patients and clinicians. In 2018, the World Health Assembly recognized the importance of DHIs for reaching the Sustainable Development Goals and recommended that these interventions be used to strengthen health systems [1-3]. Despite global support for these technologies, there is mixed evidence on their empirical benefits, cost-effectiveness, and scalability [4-7].

Low- and middle-income countries (LMICs) are beginning to introduce electronic immunization registries (EIRs) into their routine immunization services to better capture and store childhood vaccination information. EIRs are computerized tools used to collect population-based vaccination data about residents within a specific geographic area. They allow for assessing vaccination coverage by provider, vaccine, dose, age, target group, and geographic area and facilitate tracking individual vaccination histories, in addition to improving the efficiency of routine data management activities [8-10]. Especially in geographies such as Africa, where 25% of children remain unimmunized or underimmunized, before the COVID-19 pandemic, technologies that quickly identify children who are due for vaccination are important for improving vaccination coverage and ultimately morbidity and mortality owing to vaccine-preventable diseases [11]. However, an improved understanding of efficiencies created by EIRs is needed in LMICs. Finding the optimal fit between a user’s task and new technology can lead to improved efficiency, acceptability, and satisfaction among users, allowing for potential improvements in health outcomes to be realized [12].

We sought to study how 3 user workflow modifications could increase efficiencies of health care worker (HCW) activities in immunization clinics. We were interested in understanding factors influencing the time spent per activity and quantifying the added value of moving from a dual–data entry workflow, where patient information is entered into both paper-based tools and the EIR, to a completely digital workflow. Although DHIs are built to improve efficiencies, these efficiencies are not always realized; therefore, it is important to describe time utilization and study how to optimize workflows [13].

### Objective

We used an interventional pretest-posttest design time and motion study to modify workflows and measure efficiencies through human-centered design (HCD) and ergonomics methods. Our aim was to describe how activity times differed after introducing 3 workflow modifications that could potentially reduce the time needed to perform routine data entry activities and simulate a completely digital workflow. HCD has become increasingly popular in the global digital health community, as it uses rapid ideation and iteration mixed methods approaches to build technology that fits users’ needs and preferences [6,14]. HCD approaches can provide formative research needed to optimize an intervention and can help increase intervention adoption [15]. Ergonomic and human factors research are considered 2 of the main methods used to evaluate work systems and implement solutions in an effort to decrease workloads and increase patient safety [16]. Direct observation, such as a time and motion study, is a standard method in human factors research and considered to be a useful technique when studying how technology changes user workflows and tasks [17-19]. We used these methods to assess the time spent by HCWs on routine activities when using an EIR and to observe challenges with usability.

## Methods

We followed the Suggested Time and Motion Procedures (STAMP) to report our study methods [20]. These procedures aim to improve the consistency of reporting time and motion research in health informatics.

### Study Design

We designed a quantitative study within a mixed methods workflow modification project. A nonrandomized factorial observational study was conducted to test how 3 workflow modifications would affect time utilization among HCWs using the EIR at the point of care (POC) compared with baseline immunization clinic workflows. Our intent was to demonstrate changes in efficiency when moving from the existing dual–data entry workflow to the intended future paperless workflow. We used the dual–data entry workflow as our baseline, rather than a paper-based workflow, because this was a crucial transition stage introduced by the government as part of the EIR implementation plan and lasted much longer than anticipated, making it important to study its impact on time spent. This stage is important for improving trust in the data as users see the paper and digital data side by side and can gain an appreciation for how the data can help improve care. Time utilization was considered from the patient perspective, from the start to end of their time spent interfacing with an HCW during an immunization session. We hypothesized that the time spent by HCWs performing routine activities would be reduced following the introduction of the workflow modifications and that the time spent on routine activities would differ by health facility size and length of experience using the EIR. We considered various existing health information system evaluation frameworks as well as the availability of data during the study design phase. The frameworks included the Fit between Individuals, Task, and Technology framework, which describes evaluating the fit among individuals, tasks, and technologies for improved user adoption, and the Smith and Carayon ergonomics balance theory of job design for stress reduction, which expands from the Fit between Individuals, Task, and Technology framework to include physical environment and organizational conditions [12,21]. Our study was informed by these frameworks but not grounded in them.

### Study Setting

Our study was conducted in Siaya County, located in Western Kenya along Lake Victoria with a population of 993,183 people as of 2019, with most people living in a rural area [22]. According to the most recent Demographic and Health Survey in 2014, 78% of children in Siaya County were fully vaccinated [23]. At the time of this study, multiple DHI projects were being deployed across the county; some HCWs included in our study were involved in other projects.

### EIR Design and Use

The International Training and Education Center for Health (I-TECH) at the University of Washington built an EIR for the Kenya Expanded Programme for Immunization to track children’s vaccination histories and identify unimmunized or underimmunized children. I-TECH adapted a tablet-based EIR application that was originally designed and developed for Zambia’s immunization program, as users and requirements were similar across countries. For the development of the Zambia EIR application, stakeholders were brought together to develop functional and system requirements that incorporated business-process workflows, ultimately selecting the open-source OpenSRP-OpenMRS software platform (OpenSRP) [24]. The platform was updated to reflect Kenya’s recommended childhood immunization schedule, closely reflecting the standard paper-based reporting forms used by HCWs during immunization sessions. It was designed as a tablet-based POC system with web-based and offline functionality connected to a central data repository. Information on a child registered in the EIR could be viewed and edited on the web from any tablet through the system. Additional information on EIR design and deployment can be found in our qualitative study [25].

Upon the completion of training and receipt of a tablet, HCWs were expected to begin using the EIR immediately, first by retrospectively entering information from the paper-based immunization registry and then by entering the data for every child seen for immunization services thereafter. Owing to the Ministry of Health requirement of maintaining paper-based records, HCWs using the EIR completed dual–data entry, inputting patient information into both the paper-based tools and the EIR at the POC or at the end of a clinic session. It should be noted that before data collection for this study, the EIR software was upgraded, which, anecdotally, solved some of the known software bugs but slowed the system’s performance and caused it to shut down unexpectedly, which prolonged time spent during an immunization session.

### Workflow Modification Intervention Description

**Figure 1:**
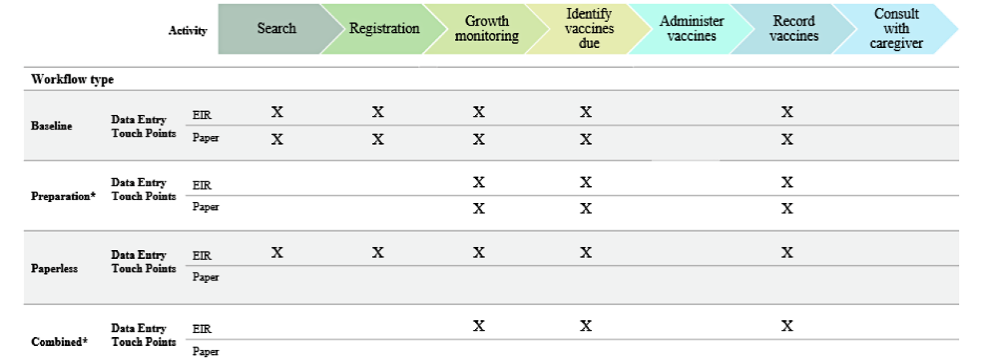
Planned data entry touch points during each immunization session activity by workflow type. EIR: electronic immunization registry. *For both the preparation and combined workflows, health care workers (HCWs) were asked to search for and manage children expected to be seen that day before the start of the immunization clinic day; therefore, they are not represented in the figure.

The baseline workflow generally encompassed a total of 7 activities for each child and varied by whether a child was due for vaccinations or needed growth monitoring, and the order of activities differed by clinic; both the EIR and paper recording tools were used concurrently at the POC (Figure 1). For the modified workflows, HCWs were trained on each data entry–related modification before the start of an immunization clinic session and then were asked to perform the modification for the length of the daily session.

The three modifications introduced were as follows:

1. Preparation: before the start of an immunization clinic session, HCWs prepared a list of children they expected to see coming in for services that day based on their next vaccination due date. Children’s names, date of birth, and EIR ID were recorded on 1 sheet of paper. The HCWs then ensured that complete data on each child on the list were preregistered in the EIR. In practice, there were 2 methods for creating the list: either the HCW gathered the home-based records from caregivers in the waiting room and wrote down each child’s information or the HCW reviewed the facility’s paper-based tools to identify which children were scheduled to come into the facility that day.
  - *Rationale*: in an effort to reduce the time it takes to search for and record information, we believed that by having HCWs gather and update information before the start of the session, they would reduce the time needed to search for and enter information during the session. We expected to reduce the time needed to identify and register a patient by batching these activities for expected children.
2. Paperless: HCWs were asked to use only the EIR during an immunization session and not paper-based tools at the POC to record information. To maintain complete records as required by Ministry of Health, HCWs entered all information collected in the EIR into the paper-based tools after the clinic session was finished.
  - *Rationale*: to simulate an ideal EIR workflow, we wanted to observe time spent by HCWs searching for and recording information when only using the EIR at the POC. The paperless workflow is the basis for how the EIR was designed and is intended to be introduced by the government in the future, when data quality is deemed to be sufficient to remove paper-based tools.
3. Combined: both of the preparation and paperless modifications described above were implemented simultaneously during a single immunization clinic session.
  - *Rationale*: we wanted to observe whether there was a synergistic effect of implementing both modifications at the same time.

### Measures

Our outcome of interest was the time taken to complete each task and immunization clinic session, comparing the time between the baseline and modified workflows. Tasks assessed included searching for a child’s record, registering a child in the EIR, identifying vaccines due, administering vaccines, growth monitoring, recording vaccines administered, and providing a consult with the caregiver. We also considered it important to assess session- and facility-specific characteristics. Session-specific characteristics included whether a patient was registered during the session and if it was their first visit, whether the child brought a home-based record (yes or no), whether any vaccines were administered (yes or no), whether 1 or >1 HCW was working at the time of observation, the number of vaccines administered during the session, EIR performance, and the clinic environment. We combined registration and first visit to create one composite categorical variable that captured whether the child was returning to the facility and had previously been registered, whether it was either a first visit or new registration, or whether it was the child’s first visit and they needed to be registered. EIR performance was captured by a composite variable that combined indicators of whether the EIR was working, partially working, or not working and whether or not it was syncing during a session; the 4 categories created were EIR working and syncing, EIR not syncing, EIR partially working but syncing, and EIR not working or syncing. For clinic environment, we created a dichotomous variable that considered a clinic to have a good environment if it was neat, uncrowded, quiet, and well lit or to have poor clinic environment if it was messy, crowded, or noisy. Facility-specific characteristics included facility type (dispensary, health center, or referral hospital), facility size (small, medium, or large, described in the next section), if adequate staff were available (yes or no), and whether the facility had <3 or ≥3 months using the EIR.

### Sampling

We collected baseline data from 12 purposively selected facilities in 3 subcounties based on their length of experience using the EIR (<3 or ≥3 months), facility size based on the 33rd and 66th percentiles of the monthly immunization target population for the county (small=≤10, medium=11-20, and large=>20), and logistical ease for data collectors. For the modified workflows, 6 facilities included in the baseline data collection with ≥3 months using the EIR, with a functional system, and located in a single subcounty were selected, as these were considered to be the facilities that could most easily accommodate the modifications owing to their experience and strong support from the subcounty.

We used a web-based computation tool for linear models to calculate the estimated sample size needed for testing a difference in time utilization between workflow types; we accounted for clustering by HCWs and workflow type [26]. The sample size calculation was performed using a significance level of *P*=.05 and 80% power. We estimated the mean values for each outcome within each group based on the EIR time-use estimates reported in the literature (Multimedia Appendix 1) [27-29]. On the basis of our specifications, our sample size was computed to be 9 HCWs, 3 per facility size. We added an additional 3 HCWs in case of attrition for a total of sample size of 12 HCWs.

At the start of data collection, few children were being seen for vaccinations daily at selected facilities; therefore, the number of child-level observations was dropped from 10 to 5 to meet the study’s timeline and not prolong the need for HCWs to perform the modified workflows. This change reduced our study power to 69%.

### Data Collection

Quantitative data were collected over the course of 2 weeks, with the first week devoted to baseline data collection and the second focused on the modified workflows. Data collectors used a standardized tool to observe and document the workflow of HCWs providing services to children seen in the immunization clinic for vaccinations or growth monitoring (data collection tools included in Multimedia Appendix 2). Data collectors were instructed to stand in the immunization room and observe an entire session, usually conducted in the morning, until at least 5 children had been observed. If <5 children were observed during one session, the facility performed the same workflow the following day and the data collectors returned the following day to complete the observations.

Each facility’s workflow was documented, including the sequence of activities, characteristics of the child being seen, whether paper tools or the EIR was used, and the number of staff working during the immunization session. Activities were timed and interruptions and other clinic observations were noted. Data collectors documented activities completed simultaneously by HCWs. Time utilization was captured from the time a child was called to receive service until they left the clinic. Data were collected on paper forms and later entered into a web-based Google Form (Google LLC).

Four data collectors were trained over 2 days on immunization program activities, use of the EIR, and how to perform observations and interviews by members of the research team (SD and RW), who also served as data collectors. Training included 1 pilot activity. All data collectors had previous experience in collecting data related to health programs. Data collectors were assigned to observe the same facility over the course of the data collection period, as much as was logistically possible, and instructed to visit the facility when it was likely to be providing immunization services.

We used the data collected during a readiness assessment completed before the deployment of the EIR for I-TECH’s project monitoring and evaluation purposes, separate from this study. All the facilities were included in the assessment in which 1 staff from each facility was interviewed about their facility’s internet connection, electricity availability, and the facility’s vaccination days. These data were collected by trained subcounty health records information officers using Google Forms or Research Electronic Data Capture (REDCap) [30,31].

### Statistical Analysis

We calculated the frequencies of facility and HCW characteristics. For the workflow observations, we used descriptive statistics to summarize activity times. The mean and SD for the amount of time to perform a given activity were calculated by workflow as well as by length of experience using the EIR (baseline only) and facility size. We also conducted bivariate testing to assess differences between workflow types and session characteristics as well as time utilization for immunization clinic activities and complete workflow time. We used an ANOVA test for unbalanced designs for continuous variables and the chi-square test for proportions. For activities that could not be timed as single events, multiple activities were timed together either because they occurred too quickly to time separately or occurred concurrently; we took the total time and divided it by the number of activities performed during that time period.

To estimate differences in time utilization between baseline and each workflow modification, we fit multivariate linear mixed-effects models. Nested random effects were included to account for the correlation between observations collected at the same facility. Fixed effects included workflow type (categorical with the baseline workflow as the reference group), EIR performance (categorical with the EIR not working or syncing as the reference group), child having a home-based record (dichotomous, yes vs no), visit and registration status (categorical with a child having been previously registered and returning to the clinic as the reference group), whether vaccines were administered (dichotomous, yes vs no), number of vaccines administered (continuous), clinic environment (dichotomous, good vs poor environment), whether >1 HCW was working at the time of observation (dichotomous, with the reference group being 1 HCW working), facility type (categorical with dispensary as the reference group), facility size (categorical with small as the reference group), whether adequate staff were available (dichotomous, yes vs no), and the number of months using the EIR (dichotomous, <3 months compared with ³3 months). Each task model included a unique set of fixed effects depending on whether the effect was relevant to the task, that is, the EIR’s performance should have no effect on administering vaccines; therefore, EIR performance was not included in that particular model.

The *Y_o_* term represents the minutes taken to complete each given task or workflow for each child observed; *B_o_X* represents the predictors, including the constant term for the mean time to complete the specific task and the workflow type for each observation. A random effect estimated the outcome of interest for each observation nested within each facility and was assumed to be normally distributed.

Yo= βoX+ uf|o

uf|o~N(0,G)

where *Y* is the time to complete task (minutes); *β* is the unknown parameters for fixed effect; *X* is the covariate vector for fixed effects; *u* is the normal (N) independent and identically distributed random effects; *G* is the variance-covariance matrix for random effects; o is the observation of fixed effect during individual child encounter in the immunization clinic; and *f* denotes facility.

All quantitative data were analyzed in R Studio (version 1.1; PBC). The *lmer* function in the R *lme4* package was used to model our linear outcomes of interest; the *lmerTest* package was used to calculate *P* values; the *lsmeans* package was used to compute contrasts for fixed effects; and the *stargazer* package was used to compile model statistics [32]. The ANOVA function in the R *car* package was used to analyze variance for unbalanced designs. Significance was determined at a 2-sided *α* value of .05.

### Ethics Approval

This study was determined to be nonhuman participant research by the University of Washington Institutional Review Board (STUDY00006256) and received human participants’ ethics approval from Amref Kenya (ESRC P587-2019), as routine program evaluation. The research team received written consent from all HCWs observed.

## Results

### Baseline Workflow Characteristics

Of the 12 facilities observed at baseline, 6 (50%) were health centers and 10 (83%) were publicly owned and administered vaccinations daily (Table 1). All 12 facilities had electricity; however, only 2 (17%) facilities had a backup power supply. Sessions were observed at 11 (92%) facilities; 1 (8%) facility had no children seen for vaccination or growth monitoring services during the study period. Of the 18 HCWs observed at baseline, 14 (78%) had been working at the facility for 1-5 years, 10 (56%) had >3 months of experience using the EIR, and 12 (67%) were nurses (Table 2).

There were 58 observations of immunization clinic sessions at baseline (Table 3). Most (55/58, 95%) children had a home-based record brought by their caregiver, and 59% (34/58) of children were previously registered in the EIR and returning to the facility for services. Only 79% (46/58) of children observed were administered a vaccination, and among these children, the mean number of vaccines administered was 2. Generally, the facility environment during the session was good (44/58, 76%), with 24% (14/58) of sessions experiencing crowding or noise or were messy, and for 59% (34/58) of the sessions, only 1 (6%) of the 18 HCWs was working in the immunization clinic. The EIR was working and syncing during 19% (11/58) of sessions, while it was not syncing during 52% (30/52) of the sessions and not working during 21% (12/58) of sessions (HCWs only used paper tools).

**Table 1:** Facility characteristics.

Characteristics		Baseline (n=12), n (%)	Modified workflow (n=6), n (%)
**Length of time using the EIR^a^ (months)**			
	<3	6 (50)	0 (0)
	≥3	6 (50)	6 (100)
**Facility type**			
	Dispensary	4 (33)	2 (33)
	Health center	6 (50)	3 (50)
	County referral hospital	2 (17)	1 (17)
**Facility size**			
	Small	4 (33)	2 (33)
	Medium	4 (33)	2 (33)
	Large	4 (33)	2 (33)
**Facility ownership**			
	Faith based	2 (17)	0 (0)
	Public	10 (83)	6 (100)
**Vaccines administered daily**			
	Yes	10 (83)	6 (100)
**Facility has electricity**			
	Yes	12 (100)	6 (100)
**Facility has backup power**			
	Yes	2 (17)	1 (17)

^a^EIR: electronic immunization registry.

**Table 2:** Health care worker characteristics.

Characteristics			Baseline (n=18), n (%)		Modified workflow (n=6), n (%)
**Years working at facility (years)**					
	<1	1 (6)		2 (33)	
	1-5	14 (78)		3 (50)	
	6-10	1 (6)		1 (17)	
	>10	2 (11)		0 (0)	
**Time spent using EIR^a^ (months)**					
	<1	0 (0)		1 (17)	
	1-3	8 (44)		0 (0)	
	≥3	10 (56)		5 (83)	
**Staff cadre**					
	Nurse	12 (67)		5 (83)	
	Nurse in-charge	3 (17)		1 (17)	
	Laboratory technician	1 (6)		0 (0)	
	Missing	2 (11)		0 (0)	

^a^EIR: electronic immunization registry.

**Table 3:** Session characteristics by workflow type.

Characteristics			Baseline (n=58), n (%)		Preparation (n=20), n (%)		Paperless (n=21), n (%)		Combined (n=27), n (%)		Overall (N=126), n (%)
**Child has home-based record (*P*=.73)**											
	Yes	55 (95)		19 (95)		19 (90)		24 (89)		117 (93)	
	No	3 (5)		1 (5)		2 (10)		3 (11)		9 (7)	
**Child visit and registration status (*P*=.61)**											
	First visit or new registration	11 (19)		4 (20)		2 (10)		2 (7)		19 (15)	
	First visit and new registration	13 (22)		4 (20)		7 (33)		5 (19)		29 (23)	
	Returning and registered	34 (59)		12 (60)		12 (57)		20 (74)		78 (62)	
**Vaccines administered (*P*=.07)**											
	Yes	46 (79)		15 (75)		12 (57)		15 (56)		88 (70)	
	No	12 (21)		5 (25)		9 (43)		12 (44)		38 (30)	
**Number of vaccines administered^a^ (*P*<.01)**											
	Values, mean (SD; range)	2 (1.5; 0-5)		2 (1.2; 0-3)		1 (1.2; 0-3)		1 (1.5; 0-4)		2 (1.5; 0-5)	
**Clinic environment^a^ (*P*=.03)**											
	Good	44 (76)		14 (70)		15 (71)		27 (100)		100 (79)	
	Messy, crowded, or noisy	14 (24)		6 (30)		6 (29)		0 (0)		26 (21)	
**EIR^b^ performance^a^ (*P*<.01)**											
	Working, but not syncing	30 (52)		9 (45)		14 (67)		22 (82)		75 (60)	
	Not working or syncing	12 (21)		0 (0)		0 (0)		0 (0)		12 (10)	
	Partially working and syncing	5 (9)		9 (45)		2 (10)		0 (0)		16 (13)	
	Working and syncing	11 (19)		2 (10)		5 (24)		5 (19)		23 (18)	
**Number of HCWs^c^ working in the clinic (*P*=.14)**											
	1 HCW	34 (59)		6 (30)		12 (57)		16 (59)		68 (54)	
	More than 1 HCW	24 (41)		14 (70)		9 (43)		11 (41)		58 (46)	

^a^Statistically significant difference between workflow types; ANOVA for unbalanced designs was used for the number of vaccines administered, and a chi-square test was used for all other variables.

^b^EIR: electronic immunization registry.

^c^HCW: health care worker.

### Modified Workflow Characteristics

The distribution of HCW characteristics during the modified workflows was similar to baseline, with most having 1-5 years of experience (3/6, 50%), ≥3 months experience using the EIR (5/6, 83%), and being nurses (5/6, 83%; Table 2). Characteristics of the children seen at a facility during the modified workflows were generally similar across workflow types, except for the number of vaccines administered, the clinic environment, and the EIR performance. Over 90% (62/69) of children had a home-based record; 57% (12/21) to 74% (20/27) of children were previously registered and returning to the facility (Table 3). Compared with baseline, fewer children were seen for vaccination during the modified workflows, ranging from 56% to 75%, and there was a significant difference in the number of vaccines administered, with those seen during the paperless and combined workflows only receiving 1 vaccination on average. The facility environment was good across each modified workflow for 70% (14/20) to 100% (27/27) of the sessions, but with significant differences; all (27/27, 100%) sessions observed during the combined workflow had a good clinic environment compared with only 76% (44/58) of sessions at baseline. The EIR was working and syncing for 19% (5/27) to 24% (5/21) of sessions; however, it was not syncing for 45% (9/20) to 82% (22/27) of sessions; there were significant differences across workflows. There were no significant differences in the number of HCWs working during the session, but there was a wide range with 41% (41/58) of sessions having >1 HCW at baseline, while 70% (14/20) of sessions for the preparation workflow had >1 HCW; during the paperless and combined workflows, only 43% (9/21) and 41% (11/27) of the sessions had >1 HCW, respectively.

### Time Utilization for Baseline Workflows

**Figure 2:**
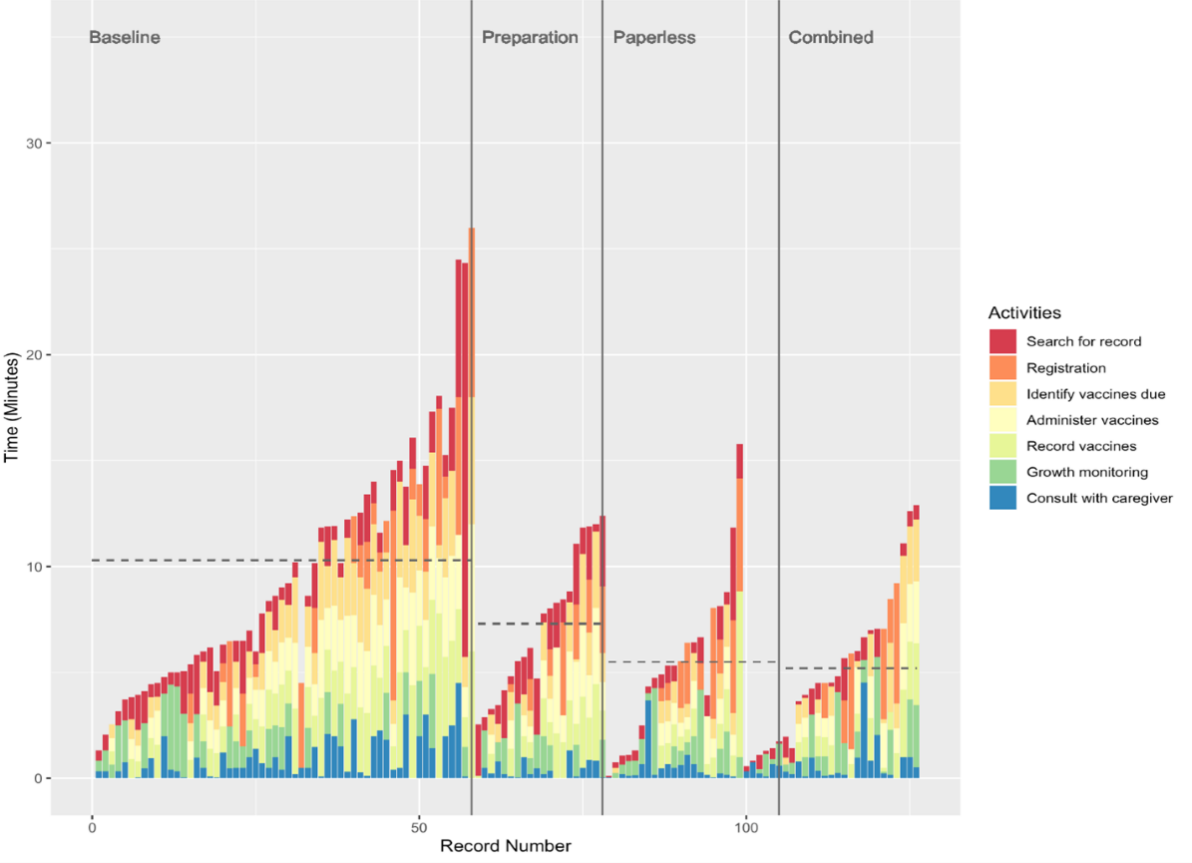
Time belt for each session by workflow type. Dashed lines indicate the mean amount of time for each workflow type.

At baseline, the mean time taken to complete a session was 10.3 minutes with an SD of 1.3 minutes (Figure 2; Table 4). Differences were observed by facility size; small facilities took 12.0 minutes per session to serve a patient, medium facilities took 9.7 minutes, and large facilities took 9.7 minutes. Those facilities with ≥3 months of experience served patients >2 minutes faster than those with <3 months experience, that is, 9.3 versus 11.4 minutes, respectively. Registration took the longest to complete (2.8 minutes), followed by administering vaccinations (2.3 minutes), recording vaccines administered (2.3 minutes), growth monitoring (2.0 minutes), and identifying vaccines due (2.2 minutes). Searching for a record and providing a consult took the least time, 1.7 and 1.01 minutes, respectively. Based on descriptive comparison of time use, it appeared that facilities with less experience typically took more time to record vaccines and providing a consult, compared with those with more experience using the EIR. On average, HCWs proportionately spent the longest amount of time on registration 22% of the total workflow time, and growth monitoring activities took 32% of their time during a single workflow (Multimedia Appendix 3).

**Table 4:** Time utilization for immunization clinic activities by workflow type and facility characteristics. The comparator group is "small facility size" for all *P* values.

Workflow type and facility characteristics				Sessions (N=126), n		Search (n=119; min)				Registration (n=46; min)				Identify vaccines due (n=83; min)					Administer vaccines (n=83; min)					Record vaccines (n=88; min)					Growth monitoring (n=75; min)					Consultation (n=113; min)					Complete workflow (min)		
						Mean (SD)		*P* value		Mean (SD)		*P* value		Mean (SD)		*P* value		Mean (SD)			*P* value		Mean (SD)			*P* value		Mean (SD)			*P* value		Mean (SD)			*P* value		Mean (SD)			*P* value
**Baseline**				58		1.7 (2.6)				2.8 (2.2)				2.2 (1.3)				2.3 (1.2)					2.3 (1.4)					2.0 (0.9)					1.1 (1.0)					10.3 (1.3)			
	**Facility size**																																								
		Small	15		3.5 (4.8)		—^a^		3.6 (1.8)		—		2.5 (0.9)		—		2.6 (0.9)			—		2.6 (0.9)			—		1.9 (0.9)			—		1.4 (1.3)			—		12.0 (7.7)			—	
		Medium	21		1.0 (0.6)		.03^b^		1.3 (0.3)		.57		2.4 (1.2)		.63		2.4 (1.2)			.89		2.4 (1.2)			.92		2.1 (1.0)			.99		1.1 (0.9)			.24		9.7 (5.4)			.49	
		Large	22		1.2 (0.6)		.06		3.7 (2.8)		.88		1.83 (1.4)		.79		2.0 (1.3)			.84		2.2 (1.7)			.63		1.6 (0.4)			.04^b^		0.7 (0.7)			.04^b^		9.7 (5.3)			.48	
	**Length of experience using the EIR^c^ (months)**																																								
		<3	28		1.6 (1.3)		—		2.5 (2.1)		—		2.0 (1.3)		—		2.2 (1.2)			—		2.4 (1.5)			—		1.9 (0.9)^d^			—		1.2 (1.2)			—		11.4 (6.0)			—	
		≤3	30		1.8 (3.5)		.96		3.9 (2.7)		.11		2.3 (1.3)		.36		2.3 (1.3)			.10		2.3 (1.3)			.06		2.0 (0.9)			<.01^d^		0.9 (0.7)			.64		9.3 (6.0)			.18	
**Preparation**				20		1.2 (0.9)		.99		1.8 (1.1)		.94		1.6 (1.0)		.22		2.0 (1.1)			.65		1.5 (1.0)			.34		1.4 (0.9)			.98		0.5 (0.4)			.07		7.3 (2.6)			.08
	**Facility size**																																								
		Small	8		1.3 (0.9)		.54		N/A^e^		—		2.2 (1.3)		.99		2.6 (1.2)			.95		2.6 (1.2)			.95		1.5 (0.4)			.55		0.7 (0.4)			.47		6.9 (3.6)			.13	
		Medium	4		1.1 (0.9)		.99		1.0 (0.9)		.58		1.7 (0.3)		.98		1.7 (0.3)			.96		1.7 (0.3)			.96		2.6 (1.2)			.63		0.3 (0.4)			.10		7.5 (3.2)			.96	
		Large	8		1.1 (2.0)		.37		2.2 (1.0)		.04^b^		0.9 (0.5)		.28		1.8 (1.2)			.93		1.0 (0.6)			.96		0.9 (0.7)			.98		0.2 (0.1)			<.01^b^		7.5 (3.4)			.93	
**Paperless**				21		0.9 (1.0)		.39		2.7 (1.9)		.99		1.0 (0.5)		<.01^f^		1.2 (0.5)			<.01^f^		1.6 (2.0)			.06		1.3 (1.2)			.99		0.5 (0.8)			.16		5.5 (0.1)			<.01^f^
	**Facility size**																																								
		Small	9		0.5 (0.2)		.21		0.7 (0.2)		.23		1.1 (0.1)		.34		1.1 (0.1)			.20		1.1 (0.1)			.18		0.8 (0.4)			.35		0.8 (1.1)			.55		3.6 (2.1)			<.01^f^	
		Medium	7		1.2 (1.6)		.62		3.6 (2.1)		.23		1.0 (0.7)		.94		1.3 (0.6)			.98		1.3 (0.6)			.99		2.6 (1.8)			.34		0.1 (0.1)			.21		5.9 (4.3)			.41	
		Large	5		1.4 (0.4)		.84		3.0 (1.6)		.06		0.8 (0.3)		.99		1.2 (0.8)			.51		2.2 (3.2)			.18		0.9 (0.3)			.99		0.5 (0.4)			.67		8.6 (4.2)			.05	
**Combined**				27		0.6 (0.5)		.09		3.4 (1.7)		.89		1.3 (1.0)		.01^f^		1.7 (0.9)			<.01^f^		1.4 (0.9)			<.01^f^		1.4 (1.2)			.99		0.7 (0.9)			.46		5.2 (0.6)			<.01^f^
	**Facility size**																																								
		Small	8		0.7 (0.4)		.27		N/A		—		1.0 (0.9)		.26		1.8 (0.6)			.36		1.3 (0.6)			.20		1.0 (0.6)			.51		1.1 (1.4)			.91		4.1 (2.5)			.01^d^	
		Medium	11		0.7 (0.4)		.87		4.0 (1.8)		.32		1.5 (1.2)		.19		2.4 (0.8)			.64		1.8 (1.3)			.49		1.8 (1.6)			.38		0.7 (0.6)			.42		7.0 (4.4)			.14	
		Large	8		0.5 (0.8)		.46^g^		2.7 (1.6)		.42		1.1 (0.3)		.04^f^		1.0 (0.3)			.99		1.0 (0.3)			.97		1.1 (0.2)			.99		0.3 (0.3)			.14		3.9 (1.7)			.02^f^	

^a^Not available.

^b^Significant difference observed compared with small facilities using an ANOVA test for unbalanced designs.

^c^EIR: electronic immunization registry.

^d^Significant difference observed between lengths of experience using an ANOVA test for unbalanced designs.

^e^N/A: not available.

^f^Significant difference observed compared with the baseline workflow using an ANOVA test for unbalanced designs.

^g^Significant difference observed compared with preparation workflow using an ANOVA test for unbalanced design.

### Time Utilization for Modified Workflows

Of the modified workflows, 85% (58/68) were typically faster than baseline, and the combined workflow was the fastest, taking 5.2 minutes to complete (Figure 2; Table 4). The preparation workflow took 7.3 minutes, and the paperless workflow took 5.5 minutes, with the paperless workflow being significantly different compared with the baseline workflow (*P*<.01). For individual activities, the time for all activities except registration was typically faster during the modified workflows compared with the baseline workflow (Table 4). There were significant differences for identifying vaccines due (*P*<.01), administering vaccines (*P*<.01), and recording vaccines (*P*<.01) for the combined or paperless workflows compared with baseline. There were some significant differences between facility sizes within each workflow but only for the baseline and preparation workflows (from *P*<.01 to *P*=–.04).

Similar to the baseline workflows, for each child seen, users proportionately spent the longest amount of time on registration and growth monitoring activities for the paperless and combined workflows, while spending more time searching for records and growth monitoring for the preparation workflow (Multimedia Appendix 3). For each modified workflow compared with baseline, improvements in the proportion of time spent on individual activities were observed for registration and growth monitoring (Multimedia Appendix 4).

### Comparisons Between Workflows

The result of the multivariate linear mixed-effects regression analysis confirmed that there were statistically significant differences in session times between the modified workflows compared with baseline, controlling for session- and facility-level characteristics (Table 5). A decrease in total session time was observed for all modified workflows, with an estimated reduction of 5.4 minutes for paperless, 4.3 minutes for combined, and 3.4 minutes for preparation workflows. Reductions in time were most frequently observed for individual activities for the paperless and combined workflows for searching for records, identifying vaccines due, recording vaccines, and growth monitoring.

For the other predictors of change in time, when the EIR was not syncing or partially working, it increased the total workflow time by 5.9 and 5.8 minutes, respectively; there were no additional significant differences when pairwise comparisons were assessed. In addition, for the total workflow time, registering a patient at their first visit increased the time by 3.1 minutes, and each additional vaccine administered increased time by 1.4 minutes, while a good clinic environment decreased the amount of time by 3.0 minutes. Health centers and county referral hospitals had increased workflow times compared with dispensaries, 10.8 and 10.2 minutes, respectively. We observed decreases in time at large- and medium-sized facilities of 15.5 and 11.4 minutes, respectively. Having adequate staffing levels increased the total workflow time by 2.9 minutes.

Across the individual activity models, results varied. There were significant reductions in time for searching for records, identifying vaccines due, recording vaccines, and growth monitoring for the paperless and combined workflows (from *P*<.01 to *P*=–.02), with the decreases in time ranging from 1.1 to 2.3 minutes. For each additional vaccine administered, the time taken to identify vaccines due, administer the vaccines, and record the vaccines increased activity times by 0.3 to 0.4 minutes, and a good clinic environment reduced the time by 1.7 and 1.5 minutes for identifying and recording vaccines, respectively.

**Table 5:** Multivariate linear mixed-effect model estimates.

				Dependent variable																																
				Amount of time per activity (min), estimated reduction (95% CI)																														Total session time, (n=124), min		
				Search (n=110)		*P* value		Registration (n=42)		*P* value		Identify vaccines due (n=81)		*P* value		Administer vaccines (n=79)		*P* value		Record vaccines (n=83)		*P* value		Growth monitoring (n=70)		*P* value		Consultation (n=105)		*P* value			Estimated reduction (95% CI)			*P* value
Constant				2.0 (−1.9 to 5.8)		.71		−1.8 (−9.5 to 5.9)		.56		3.3 (1.1 to 5.6)		.02^a^		2.8 (1.6 to 3.9)		.02^a^		2.4 (0.4 to 4.4)		.02^a^		−3.1 (−6.0 to −0.2)		.28^a^		1.1 (−0.4 to 2.5)		.30			6.0 (0.4 to 11.6)			.04^a^
**Workflow type (compared with baseline)**																																				
	Modification—preparation			−1.6 (−3.2 to 0.0)		.02		−0.9 (−4.4 to 2.5)		.61		−0.8 (−1.7 to 0.1)		.07		−0.1 (−0.8 to 0.7)		.07		−0.5 (−1.5 to 0.5)		.07		−2.0 (−3.0 to −1.0)		<.01^a^		−0.8 (−1.5 to −0.2)		.01^a^			−3.4 (−5.9 to −0.9)			.01^a^
	Modification—paperless			−1.8 (−3.2 to −0.4)		.02^a^		−2.5 (−5.5 to 0.5)		.25		−2.3 (−3.1 to −1.4)		<.01^a^		−1.2 (−2.0 to −0.4)		<.01^a^		−2.1 (−3.1 to −1.1)		<.01^a^		−1.2 (−1.8 to −0.6)		<.01^a^		−0.5 (−1.0 to 0.0)		.05			−5.4 (−7.6 to −3.3)			<.01^a^
	Modification—combined			−1.6 (−2.8 to −0.4)		.01^a^		−2.4 (−6.0 to 1.2)		.39		−1.3 (−2.0 to −0.6)		<.01^a^		−0.5 (−1.3 to 0.2)		<.01		−1.4 (−2.3 to −0.6)		<.01^a^		−1.1 (−1.6 to −0.6)		<.01^a^		−0.2 (−0.6 to 0.3)		.43			−4.3 (−6.3 to −2.4)			<.01^a^
**Session characteristics**																																				
	EIR^b^ not syncing (compared with not working)			0.3 (−1.5 to 2.1)		.80		3.9 (1.6 to 6.1)		.02^a^		0.3 (−0.7 to 1.2)		.55		N/A^c^		—^d^		0.9 (−0.2 to 2.0)		.55		1.1 (−0.0 to 2.2)		.05		0.3 (−0.5 to 1.1)		.39			5.9 (2.9 to 8.8)			<.01^a^
	EIR partially working (compared with not working)			0.6 (−1.7 to 2.9)		.71		1.8 (−2.8 to 6.3)		.47		−0.6 (−1.8 to 0.7)		.39		N/A		—		−0.1 (−1.3 to 1.1)		.39		2.2 (0.5 to 3.9)		.02^a^		1.0 (0.1 to 1.9)		.05			5.8 (2.6 to 9.0)			<.01^a^
	EIR working (compared with not working)			−0.4 (−3.0 to 2.1)		.70		1.1 (−3.3 to 5.5)		.60		−1.2 (−2.6 to 0.2)		.09		N/A		—		−0.8 (−2.5 to 0.9)		.09		−0.4 (−1.8 to 1.0)		.63		0.3 (−0.7 to 1.3)		.57			3.6 (−0.5 to 7.6)			.08
	Child has home-based record			−0.7 (−3.0 to 1.6)		.21		0.3 (−1.9 to 2.4)		.64		−0.7 (−1.7 to 0.3)		.20		N/A		—		−0.5 (−1.6 to 0.7)		.20		0.7 (−0.5 to 1.8)		.59		−0.1 (−1.0 to 0.7)		.79			−0.9 (−4.2 to 2.4)			.59
	First visit, with registration (compared with returning and registered patient)			0.7 (−0.6 to 1.9)		.99		N/A		—		0.8 (0.2 to 1.4)		.01^a^		N/A		—		0.7 (0.0 to 1.5)		.01		0.6 (−0.2 to 1.4)		.12		0.1 (−0.4 to 0.5)		.95			3.1 (1.2 to 5.0)			<.01^a^
	First visit or new registration (compared with returning and registered patient)			0.7 (−0.8 to 2.1)		.42		N/A		—		0.5 (−0.2 to 1.2)		.20		N/A		—		0.7 (−0.2 to 1.5)		.20		−0.4 (−1.3 to 0.5)		.69		−0.2 (−0.8 to 0.5)		.59			2.1 (−0.3 to 4.4)			.09
	Vaccines administered			N/A		—		N/A		—		−0.4 (−1.6 to 0.8)		.49		N/A		—		N/A		—		N/A		—		N/A		—			1.6 (−1.1 to 4.3)			.24
	Number of vaccines administered			N/A		—		N/A		—		0.3 (0.1 to 0.6)		<.01^a^		0.3 (0.0 to 0.5)		.01^a^		0.4 (0.1 to 0.7)		.01^a^		N/A		—		N/A		—			1.4 (0.6 to 2.2)			<.01^a^
	Good clinic environment (compared with poor)			−0.8 (−2.5 to 0.9)		.79		0.1 (−3.8 to 3.9)		.91		−1.7 (−2.6 to −0.7)		<.01^a^		−0.9 (−1.7 to −0.1)		<.01		−1.5 (−2.5 to −0.6)		.01^a^		−0.9 (−2.2 to 0.3)		.05		−0.1 (−0.7 to 0.6)		.82			−3.0 (−5.4 to −0.6)			.02^a^
	>1 HCW^e^ in workflow (compared with 1 HCW)			−0.5 (−1.7 to 0.7)		.64		−0.0 (−2.3 to 1.5)		.81		−0.7 (−1.4 to 0.0)		.04^a^		−0.6 (−1.2 to 0.1)		.05		−0.8 (−1.5 to 0.0)		.05		0.3 (−0.4 to 1.0)		.50		0.2 (−0.3 to 0.7)		.44			−0.4 (−2.3 to 1.5)			.68
**Facility characteristics**																																				
	**Facility type**																																			
		Health center (compared with dispensary)	−0.2 (−4.1 to 3.8)		.70		0.7 (−8.4 to 9.8)		.97		2.0 (0.0 to 4.1)		.13		1.3 (−0.2 to 2.9)		.13		2.1 (0.0 to 4.1)		.13		−0.9 (−3.5 to 1.6)		.95		1.0 (−0.6 to 2.6)		.33			10.8 (5.6 to 16.1)			<.01^a^	
		County referral hospital (compared with dispensary)	0.2 (−4.0 to 4.4)		.95		2.2 (−8.3 to 12.8)		.85		1.0 (−1.2 to 3.2)		.42		0.5 (−1.1 to 2.2)		.42		1.6 (−0.4 to 3.7)		.42		0.1 (−2.3 to 2.4)		.61		1.8 (0.1 to 3.5)		.09			10.2 (4.6 to 15.8)			<.01^a^	
	**Facility size**																																			
		Large (compared with small)	−1.2 (−5.8 to 3.3)		.95		−0.8 (−12.1 to 10.5)		.97		−3.2 (−5.6 to −0.8)		.08		−1.7 (−3.4 to 0.1)		.08		−3.6 (−5.9 to −1.4)		.07^a^		−0.7 (−3.0 to 1.7)		.24		−2.0 (−3.8 to −0.2)		.10			−15.5 (−21.5 to −9.5)			<.01^a^	
		Medium (compared with small)	−0.8 (−4.7 to 3.2)		.91		−0.2 (−9.0 to 8.6)		.94		−1.9 (−4.0 to 0.3)		.18		−1.2 (−2.7 to 0.3)		.18		−2.3 (−4.2 to −0.3)		.18		1.7 (−0.8 to 4.1)		.64		−1.4 (−3.0 to 0.2)		.19			−11.4 (−16.4 to −6.4)			<.01^a^	
	Adequate staff available			1.1 (−1.0 to 3.3)		.35		0.6 (−4.8 to 6.0)		.80		0.9 (−0.3 to 2.2)		.23		−0.1 (−1.1 to 0.9)		.23		1.2 (−0.1 to 2.4)		.23		2.3 (1.5 to 3.2)		<.01^a^		0.4 (−0.4 to 1.2)		.34			2.9 (0.6 to 5.3)			.02^a^
	>3 months using EIR (compared with <3 months)			1.6 (−1.1 to 4.3)		.24		2.2 (−2.9 to 7.4)		.53		0.9 (−0.5 to 2.3)		.25		0.1 (−0.8 to 0.9)		.25		0.5 (−1.0 to 2.0)		.25		3.5 (1.7 to 5.2)		<.01^a^		−0.2 (−1.2 to 0.8)		.86			−1.8 (−4.7 to 1.1)			.22

^a^Statistically significant at a 2-sided *α* value of .05.

^b^EIR: electronic immunization registry.

^c^N/A: not applicable.

^d^Not available.

^e^HCW: health care worker.

## Discussion

### Principal Findings

Our study found that for HCWs using an EIR, the introduction of modified workflows decreased the amount of time needed to provide services to children seen in the immunization clinic. The prolonged use of dual–data entry workflow is not ideal from a user or program perspective but is a mechanism to ensure that immunization records are maintained while the EIR’s reliability is tested. This study provides evidence for ensuring the reliability of an EIR as quickly as possible and allowing facilities to move to a paperless workflow. With a baseline mean time of 10 minutes spent per child, this decreased by about 3 minutes when the preparation modification was introduced and almost 5 minutes for the paperless and combined modifications. Our results further demonstrate the necessity of modifying immunization clinic workflows upon DHI introduction to increase the efficiency by fitting workflows to specific clinic settings and adapted for HCW use cases.

Our initial hypothesis that there would be differences seen at baseline by size of facility and length of experience was confirmed. At baseline, larger facilities and those with more experience using the EIR tended to serve patients faster (although not statistically significant), as would be expected if we assumed increased experience, either by patient volume or length of time using the EIR, which would lead to more efficient workflows. Differences were also observed during the modified workflows; however, the trends were reversed for the preparation and paperless workflows, where larger facilities took more time to complete activities, while medium-sized facilities took the longest for the combined workflow. Our model estimates indicated large time utilization differences, in opposite directions, with health centers and referral hospitals having longer times, while large- and medium-sized facilities had shorter times, despite these characteristics being related. Although this warrants further investigation, we hypothesize that facility size better reflects efficiencies created by high patient volumes and possibly more staff, while facility type is strictly a government designation that could categorize facilities of varying capacity together.

Our expectation that the paperless workflow would decrease the total workflow time was realized, as this was the only workflow where a single data source was used throughout an immunization session. This further emphasized that users should switch to the intended future paperless workflow once managers are satisfied with EIR data quality and performance and have proper guidance in place. We also observed a small synergistic effect for the combined workflow, leading us to conclude that the optimal workflow is paperless with a child having complete and up-to-date information in the EIR. We also conducted a qualitative study as part of this project to understand the major barriers and facilitators to EIR use among HCWs; based on our qualitative findings, there were no differences observed in users’ perceptions of the combined workflow compared with the others [33]. Our finding that larger facilities took more time than small facilities to complete immunization sessions for the modified workflows could possibly be due to the added complexities of introducing workflow changes into already busy or crowded settings, where it may take more time to adapt to a change when other environmental factors are at play. In addition, our finding that sessions with adequate staffing levels increased workflow times was counterintuitive, as we would have expected times to decrease; however, this could potentially be due to facilities having preexisting limited staffing levels that were anecdotally noted to strain clinic staff.

Results pertaining to the EIR’s performance and ability to connect to the internet were particularly insightful about potential causes of delays. Facilities with poor internet access may have experienced delays when the EIR tried to sync records stored in the central server, subsequently causing workflow time to increase and may have led to the large variability that we observed in workflow time. When the EIR was fully functional and syncing with the server, activities took less time. Our qualitative study found that HCWs felt more time pressure and frustration, that more effort was required when there were connectivity issues, and that these feelings were exacerbated when there were many patients to be seen or staffing shortages [25].

Our study was guided by multiple DHI-related theories and those data that we could readily collect. These theories provided meaningful structure for designing data collection instruments, and our findings reinforced the importance of studying the linkages between individuals, tasks, and technology, as well as taking into consideration the broader environmental and organization context. Our qualitative paper describes the underlying mechanisms linking workflow processes to outcomes in more detail [25].

### Time Savings

This study highlights where areas of potential time savings can be found for immunization clinics using an EIR. In addition to improving EIR performance at the POC to save time, alternative mechanisms for registering children in the EIR should be explored. In terms of session-specific efficiency gains, additional time savings could be observed if facilities were willing to change the order patients were seen for services; for instance, in a clinic with 2 nurses, if patients needing to be registered were attended to by 1 HCW while preregistered children were seen by another HCW for vaccinations, potentially time could be saved, but this would need to be tested. Future studies could consider quantifying EIR and internet performance to determine when to expect time savings and create a minimum performance standard that could be used to help decision makers decide when and whether this type of technology should be introduced as a paperless alternative to paper-based records. Projecting time savings and subsequent cost savings would be important for demonstrating the value of the system.

There are no other published studies that have specifically assessed time utilization between a dual–data entry workflow and a paperless workflow; however, there are evaluation reports in the gray literature of time utilization following the introduction of an EIR implemented in Afghanistan, the Gambia, and Uganda comparing paper-based with paperless data management activities [27-29]. In each country, reductions in time utilization for a child’s first immunization visit were observed, ranging from 1.45 to 6.21 minutes. The authors estimated that in Afghanistan the EIR would save US $2.9 million over 5 years, which would be US $0.40 per child, based on the value of the time saved from completing data administration tasks, and US $2.1 million, US $0.28 per child, in Uganda.

Numerous studies have estimated the time savings of implementing electronic health record systems, with varied results. A systematic review of the impact of these systems on documentation time found that when physicians and nurses used bedside terminals and central station desktops, they saved around 24% of their overall time spent documenting during their shift, but when they used POC systems, their documentation time increased by 18% [13]. However, a study of an electronic medication management system found no significant change in the proportion of time clinicians spent on direct care or medication-related tasks [34]. Furthermore, evaluations conducted soon after the introduction of a technology initially observed reductions in documentation time; however, increased documentation time was observed when a longer period of time had passed between introduction and evaluation [34].

### Future Research

In light of our study’s findings and the discrepancies in time savings found in the literature, there is further need for DHI researchers to use methodology that assists with understanding the relationship between intervention innovation and service innovation [35]. Both HCD and implementation science methods need to be used together to better understand this relationship in which HCD methods are used to study an intervention’s acceptability in a laboratory-based setting, while implementation science methods aim to understand whether an intervention is effective in a health care delivery setting. Other DHI researchers have pointed out that taking a service design approach that explicitly acknowledges how new interventions need to be adapted to fit their setting can bridge the gap between methodologies and researchers should in fact be evaluating the interaction between a DHI and established health care service delivery routines [35]. Conducting clinical simulations, similar to our study, can provide researchers a low-cost approach to evaluating DHI in complex health care systems and generate evidence needed between formative and large-scale implementation stages [36].

Although time use was our study’s outcome of interest, quantifying time savings may not always be the best metric for assessing the impact of a DHI. Time savings may be realized within a well-functioning health care system with adequate resources; however, for systems lacking these assets, DHI may increase the amount of time needed to perform routine tasks because they add complexity to HCW duties. Time is a finite resource that has implications for budgeting and reaching every child in need of health services, but measuring time may not be the ideal metric when attempting to improve the quality of health care services. Monitoring changes in data quality to understand the accuracy and completeness of records or how time is used to improve service quality, such as measuring whether caregiver consultations cover all recommended topics, could be alternative metrics. Researchers studying DHI should be encouraged to measure intermediate metrics over the course of an intervention’s introduction and scale-up to understand whether the DHI is achieving high fidelity before assessing efficiencies and impact.

We sought to examine the use of time for patient-facing activities during an immunization session; this was used as a proxy for overall client time in the facility because we did not measure patient waiting time. Typically, DHIs seek to maximize patient time with a provider, while minimizing total time to seek services. Our study of patient time was focused on whether the workflow modifications could reduce the total session time and whether time to complete data management activities could be reduced. Because we did not design this study to maximize patient time with a provider, future interventions should consider how workflows can be modified to repurpose time used for data management activities into time used for patient-facing consultation.

### Study Strengths

We were able to conduct a quick clinical simulation exercise of introducing modified workflows and estimating their impact on time utilization in immunization clinics using an EIR. This study demonstrated the necessity of assessing and incorporating contextual factors to adequately understand the impact of a new technology on a health care setting in an LMIC. In addition, the study provided pragmatic and policy-relevant evidence in support of the paperless data entry workflow being efficient once the EIR performance and internet connectivity issues were solved.

### Limitations

This study has several limitations. Our sample size was halved because of the unexpectedly few children that visited immunization clinics daily, far below what routine health information system data estimated. Due to the nature of performing time and motion observations, this study potentially could have suffered from the Hawthorne effect because data collectors were required to stand in the immunization room, and their presence could have influenced how the HCWs performed their tasks. Our study suffered from low fidelity of the EIR owing to unexpected issues with the platform’s performance and intermittent internet connectivity. Also, as this was a cross-sectional assessment, we were not able to assess how HCWs become more familiar with the workflow modifications. Activity times had lower than ideal precision owing to difficulty with capturing activities that occurred quickly in sequence or were simultaneous. The use of purposive sampling could have potentially introduced bias because it was nonrandomized and facilities selected may not provide representative results.

### Conclusions

Using a time and motion study, we were able to demonstrate the necessity of modifying immunization clinic workflows to actualize value when introducing an electronic system. We found that the paperless workflow provided the largest time savings when delivering services, although this was threatened by poor EIR performance and internet connectivity. This study demonstrated the benefit of evaluating a DHI in different settings to better understand and find the best fit between user tasks and technology, ultimately demonstrating that not only should DHIs be built and adapted for particular use cases but that existing user workflows also need to adapt to new technology.

## Appendix

Multimedia Appendix 1 Workflow time estimates for sample size calculations.

Multimedia Appendix 2 Data collection tools.

Multimedia Appendix 3 Mean proportion of time spent on each activity for an individual workflow.

Multimedia Appendix 4 Difference in mean proportion time used between baseline and each modified workflow.

## Abbreviations

DHI: digital health intervention
EIR: electronic immunization registry
HCD: human-centered design
HCW: health care worker
I-TECH: International Training and Education Center for Health
LMIC: low- and middle-income country
POC: point of care
REDCap: Research Electronic Data Capture
STAMP: Suggested Time and Motion Procedures
