# Supplementary material for: Time Utilization Among Immunization Clinics Using an Electronic Immunization Registry (Part 2): Time and Motion Study of Modified User Workflows
Source: JMIR Form Res. 2023 Mar 16;7:e39777. doi: 10.2196/39777 (PMC10019767; doi:10.2196/39777)
Supplement: Multimedia Appendix 1 [file formative_v7i1e39777_app1.docx]

**Appendix Table 1. Workflow Time Estimates for Sample Size Calculations**

| **Workflow** | **1 (Baseline)** | **2 (Workflow Redesign 1)** | **3 (Workflow Redesign 2)** |
| --- | --- | --- | --- |
| **Facility Size** |  |  |  |
| **Small** | 8 min | 7 min | 7 min |
| **Medium** | 6 min | 5 min | 4 min |
| **Large** | 5 min | 4 min | 4 min |

We used an online computation tool for linear models to calculate the estimated sample size needed for testing a difference in time utilization between workflow types; we accounted for clustering by HCW and workflow type [27]. The sample size calculation was performed using a significance level of 0.05 and 80% power. To account for clustering at the HCW level, we estimated an intracluster correlation of 0.8 for all observations from each HCW and assumed that we would observe each HCW vaccinate 10 children. The size of a facility (categorical) was entered as a between-participant factor (fixed predictor) as we wanted to assess changes in time by facility size, we assumed that there would be an equal number of HCWs observed in each category. We estimated the mean values for each outcome within each group based on the EIR time-use estimates reported in the literature (see Appendix Table 1)[28-30]. We expected a correlation of 0.5 between workflow types for each HCW, and a constant standard deviation across observations of 1 minute. Based on our specifications, our sample size was computed to be 9 HCWs, 3 per facility size. We added an additional 3 HCWs in case of attrition, for a total of sample size of 12 HCWs.
