# Supplementary material for: Time Utilization Among Immunization Clinics Using an Electronic Immunization Registry (Part 2): Time and Motion Study of Modified User Workflows
Source: JMIR Form Res. 2023 Mar 16;7:e39777. doi: 10.2196/39777 (PMC10019767; doi:10.2196/39777)
Supplement: Multimedia Appendix 3 [file formative_v7i1e39777_app3.docx]

**Appendix Table 3. Mean proportion of time spent on each activity for an individual workflow**

| **Workflow Type** | **Facility Characteristic** | **Number of Workflows** | **Search** | **Registration** | **Identify vaccines due** | **Administer vaccines** | **Record vaccines** | **Growth monitoring** | **Consult** |
| --- | --- | --- | --- | --- | --- | --- | --- | --- | --- |
| **Baseline** | **All** | 58 | 18% | 22% | 19% | 20% | 21% | 32% | 11% |
|  |  |  |  |  |  |  |  |  |  |
|  | **Small** | 15 | 28% | 32% | 17% | 17% | 17% | 29% | 13% |
|  | **Medium** | 21 | 14% | 11% | 21% | 21% | 21% | 36% | 12% |
|  | **Large** | 22 | 16% | 28% | 19% | 21% | 22% | 25% | 10% |
|  |  |  |  |  |  |  |  |  |  |
|  | **< 3 months using EIR** | 28 | 17% | 20% | 19% | 20% | 21% | 24% | 10% |
|  | **>= 3 months using EIR** | 30 | 20% | 31% | 20% | 20% | 20% | 34% | 12% |
| **Preparation** | **All** | 20 | 25% | 24% | 20% | 23% | 20% | 29% | 7% |
|  |  |  |  |  |  |  |  |  |  |
|  | **Small** | 8 | 24% |  | 26% | 26% | 26% | 38% | 12% |
|  | **Medium** | 4 | 18% | 14% | 22% | 22% | 22% | 43% | 3% |
|  | **Large** | 8 | 30% | 28% | 13% | 22% | 16% | 14% | 3% |
| **Combined** | **All** | 27 | 19% | 49% | 19% | 24% | 19% | 30% | 20% |
|  |  |  |  |  |  |  |  |  |  |
|  | **Small** | 8 | 23% |  | 16% | 30% | 22% | 24% | 30% |
|  | **Medium** | 11 | 21% | 49% | 18% | 23% | 16% | 26% | 19% |
|  | **Large** | 8 | 11% | 50% | 26% | 22% | 22% | 42% | 11% |
| **Paperless** | **All** | 21 | 23% | 32% | 17% | 17% | 18% | 33% | 14% |
|  |  |  |  |  |  |  |  |  |  |
|  | **Small** | 9 | 21% | 14% | 20% | 20% | 20% | 36% | 22% |
|  | **Medium** | 7 | 30% | 40% | 19% | 14% | 14% | 50% | 7% |
|  | **Large** | 5 | 15% | 35% | 12% | 17% | 19% | 11% | 8% |
|  |  | 126 | 119 | 46 | 83 | 83 | 88 | 75 | 113 |
