# Supplementary material for: Time Utilization Among Immunization Clinics Using an Electronic Immunization Registry (Part 2): Time and Motion Study of Modified User Workflows
Source: JMIR Form Res. 2023 Mar 16;7:e39777. doi: 10.2196/39777 (PMC10019767; doi:10.2196/39777)
Supplement: Multimedia Appendix 4 [file formative_v7i1e39777_app4.docx]

**Appendix Table 4. Difference in Mean Proportion Time Used between Baseline and Each Modified Workflow**

| **Workflow Type** | **Facility Characteristic** | **Search** | **Registration** | **Identify vaccines due** | **Administer vaccines** | **Record vaccines** | **Growth monitoring** | **Consult** |
| --- | --- | --- | --- | --- | --- | --- | --- | --- |
| **Preparation** | **All** | 7% | 2% | 1% | 3% | -1% | -2% | -4% |
|  |  |  |  |  |  |  |  |  |
|  | **Small** | -4% | -32% | 8% | 9% | 9% | 9% | -1% |
|  | **Medium** | 4% | 4% | 2% | 2% | 2% | 7% | -8% |
|  | **Large** | 14% | 0% | -5% | 1% | -7% | -11% | -6% |
| **Combined** | **All** | 1% | 27% | 0% | 4% | -1% | -2% | 9% |
|  |  |  |  |  |  |  |  |  |
|  | **Small** | -5% | -32% | -1% | 13% | 5% | -5% | 17% |
|  | **Medium** | 8% | 38% | -3% | 2% | -4% | -10% | 7% |
|  | **Large** | -5% | 22% | 8% | 1% | 0% | 17% | 1% |
| **Paperless** | **All** | 4% | 10% | -2% | -3% | -3% | 1% | 3% |
|  |  |  |  |  |  |  |  |  |
|  | **Small** | -8% | -18% | 3% | 3% | 3% | 7% | 9% |
|  | **Medium** | 16% | 30% | -2% | -7% | -7% | 14% | -5% |
|  | **Large** | -1% | 7% | -7% | -4% | -4% | -14% | -1% |
